# Prognostic Value of Serum Osteoprotegerin Level in Patients With Hepatocellular Carcinoma Following Surgical Resection

**DOI:** 10.3389/fonc.2021.731989

**Published:** 2021-09-28

**Authors:** Chihao Zhang, Jiayun Lin, Xiaochun Ni, Hongjie Li, Lei Zheng, Zhifeng Zhao, Xiaoliang Qi, Haizhong Huo, Xiaolou Lou, Qiang Fan, Meng Luo

**Affiliations:** Department of General Surgery, Shanghai Ninth People’s Hospital, School of Medicine, Shanghai Jiao Tong University, Shanghai, China

**Keywords:** hepatocellular carcinoma, osteoprotegerin, RANKL, overall survival, disease-free survival

## Abstract

**Background:**

Multiple studies have reported that tissue or serum osteoprotegerin (OPG) level is a prognostic factor for patients with cancer. However, little is known about the role of serum OPG in hepatocellular carcinoma (HCC). In this study, we aimed to investigate whether serum OPG concentration has an effect on HCC patients’ prognosis.

**Methods:**

A total of 386 eligible HCC patients undergoing radical hepatectomy were enrolled from Shanghai Ninth People’s Hospital and Zhongshan Hospital between 2010 and 2018. Kaplan-Meier curves, Cox regression model, and the restricted mean survival time (RMST) were used to estimate the association of OPG and HCC patients’ survival outcome. In addition, sensitivity analyses were carried out including subgroup analysis and propensity score matching (PSM).

**Results:**

Patients were separated into two groups according to the cut-off value of OPG calculated by X-tile. Multivariate Cox analysis showed that patients with high OPG level had worse overall survival (OS) (HR: 1.93; 95% CI: 1.40–2.66, p<0.001) and disease-free survival (DFS) (HR: 1.85; 95% CI: 1.39–2.47, p<0.001) before matching. On average, RMST ratio between high and low OPG turned out to be 0.797 (95% CI: 0.716–0.887, p<0.001). In the matched population, we found that OPG level was negatively associated with OS (HR: 1.85; 95% CI: 1.25–2.74, p=0.002) and DFS (HR: 1.71; 95% CI: 1.20–2.44, p=0.003). In addition, a similar trend was further confirmed by subgroup analyses.

**Conclusion:**

In a word, HCC patients with high OPG level had poorer survival rates compared with HCC patients with low OPG level. This factor could act as a potential prognostic predictor for HCC patients who underwent radical resection in the future.

## Introduction

It is reported that the incidence of hepatocellular carcinoma (HCC) ranks sixth along with the mortality rate ranking third among all malignant cancers worldwide. Specifically, it has been estimated that there were approximately 841,000 newly diagnosed HCC cases, and 782,000 died from this cancer every year (1). Although the pattern of HCC varies according to the different regions or counties, more than 50% emerging HCC patients in the world are from China (2–4). Unfortunately, despite obvious improvements in treatment, patients with HCC still have shorter survival time compared with other cancers. Because quite a large proportion of patients miss the opportunity to receive curative therapies, such as surgical removal, liver transplantation, or radiofrequency ablation (RFA), due to the fact that patients with HCC have no symptoms in its early stages and are discovered late at an initial diagnosis (5, 6). Therefore, it is necessary to find appropriate prognostic biomarkers to stratify patients’ survival and determine who could benefit from the therapies.

So far, some clinical factors, such as alpha-fetoprotein (AFP), neutrophil-to-lymphocyte ratio (NLR), and serum gamma-glutamyl transpeptidase (GGT), have been put forward for the early detection, risk prediction, and monitoring use of HCC (7–9). Among them, AFP was most studied and was reported by Japanese scholars that it possessed abilities to predict HCC patients’ prognosis following liver surgery (10). However, it remains controversial regarding the sensitivity of AFP, which limits its clinical value. It is estimated that serum AFP is significantly elevated in 2/3 HCC patients, which means nearly 1/3 patients have normal AFP level, especially when the tumor is in early stages. Under this circumstance, AFP is not an ideal candidate for prognostic prediction of HCC if it falls into false negativeness.

In recent years, the role of inflammatory factors in HCC has aroused much attention, considering its tight association with tumor development, including initiation, proliferation, angiogenesis, and metastasis (11, 12). Osteoprotegerin, a member of tumor necrosis factor receptor (TNFR) family, can suppress RANK-signaling by binding to RANKL and then stimulate osteoclast maturation (13). Subsequently, researchers discovered that OPG was also expressed by different cancers, including breast cancer, prostate cancer, colorectal cancer, and so on. It has shown that OPG can prolong prostate cancer cells’ life *in vitro* by inhibiting TRAIL-dependent apoptosis (14, 15). In contrast, when prostate cancer cells metastasize to the bone, OPG was found to have antitumor abilities. In addition, it has shown that OPG was overexpressed in pancreatic cancer tissues compared with normal ones and was associated with poor survival outcome as well as the occurrence of diabetes mellitus (16). Despite these findings, we know little about the role of serum OPG in HCC, and so it deserves further investigation. Therefore, in this study, we aimed to explore whether preoperative serum OPG levels were correlated with survival time of HCC patients after surgical resection.

## Materials and Methods

### Patients

Eligible patients from Shanghai Ninth People’s Hospital and Shanghai Zhongshan Hospital were collected in our retrospective study between 2011 and 2017 according to the following inclusion criteria: (1) patients with primary HCC confirmed pathologically; (2) underwent radical hepatectomy; (3) no evidence of distant metastasis; (4) no history of other malignant tumors; (5) without preoperative systemic or local therapies. Patients who were followed up less than 1 month or had no complete clinicopathological information are not allowed into further analysis. In the end, a total of 386 patients met the above criteria and were included in this study. This study was approved by the Ethics Committee of the above hospitals. As far as we know, all these patients did not undergo active inflammatory or autoimmune disease, dental problems, and bone-related diseases when they were admitted into the hospital.

The clinicopathological variables were retrospectively collected from medical records and laboratory tests, including age, gender, alanine aminotransferase (ALT), total bilirubin (TB), albumin, AFP, γ-glutamyl transpeptidase (GGT), and hepatitis B serology. All laboratory parameters were tested 3 days before surgery. In addition, tumor-related characteristics like tumor size, number, encapsulation, and microvascular invasion were acquired by imaging or pathological examination. The clinical stage of each patient was determined according to the Tumor Node Metastasis (TNM) staging system of AJCC 8^th^ edition. Each patient was monitored periodically after operation with interval time of 3 months in the first 2 years, followed by once a year thereafter. The tests consisted of serum AFP, abdominal CT or ultrasound, and blood biochemistry examination.

The main endpoints in this study were OS and DFS. OS was referred to the death of any cause, including individuals who died of other non-tumor-related causes. While DFS was defined as a survival measure representing the survival without recurrence after surgical removal.

### Serum Samples

Ten milliliters of blood samples were taken from each patient with an S-Monovette (Sarstedt AG &Co.) once the patient was confirmed as HCC before surgery and then kept in −4°C. To avoid blood cell lysis, the samples were delivered to the central laboratory within 30 min after blood drawing and must be dealt immediately within 4 h. Subsequently, 3–4 ml of supernatant constituted of blood serum was obtained after centrifugation at 2,000 g for 10 min and stored at −80°C. When we collected all the blood serum samples, we started to measure those frozen samples using ELISA at the same time.

### Measurement of Serum OPG by ELISA

OPG concentrations in serum were determined strictly following the instructions of enzyme-linked immunosorbent assay (ELISA) kit (Sigma, USA). The Thermoscientific Varioskan Flash microplate reader (Waltham, MA, USA) was used to measure absorbance of OPG at 450 nm with 630 nm as a reference. Serum samples from a patient were assayed in duplicate on the plate. Therefore, OPG level was equal to the average value of these duplicate samples.

### Statistical Analysis

The baseline information of the HCC patients was listed with descriptive statistics. Absolute number with proportion was used to describe categorical variables. X-Tile software was used to confirm the optimal cut-off value of OPG for overall survival prediction in this study. The survival curves were plotted using Kaplan-Meier method to show OS and DFS with log-rank test used for prognostic comparison between groups. To explore potential variables associated with OS or DFS, univariate Cox regression model was applied. Any significant variables (P <0.05) were allowed to be included in multivariate Cox analysis with HR and its 95% confidence intervals as risk estimates. However, HR may not a reliable indicator to measure the real effects when the assumption of proportional hazards suffers violation. Thus, the restricted mean survival time (RMST) was calculated and used to compare the survival time of HCC patients in different groups. Furthermore, we adjusted the RMST with the same covariates identified in the Cox regression model. In addition, the ratio of RMSTs between OPG high and low groups was calculated to compare survival difference. The ratio <1 means the patients are not able to obtain survival benefit from high serum OPG level.

As this, in essence, is a retrospective study, the potential covariates were not well balanced between OPG high and low groups. It may distort OPG’s real relationship with OS and DFS. To reduce this influence, PSM was used to perform a matched case-control analysis. First, each patient will be allocated a propensity score which was calculated using logistic regression modeling with OPG as a dependent variable. The remaining confounding variables listed in **Table 1** were taken as covariates. Second, patients in OPG high and low groups were matched at 1:1 fixed ratio if they had similar propensity scores. The method we used in this process is called nearest available matching with the caliper of 0.05. Third, standard difference (SD), acted as an indicator of the matching effect, was calculated for all the clinical variables. The value <0.1 indicated that the covariates are well distributed between two groups. Fourth, after matching, we plotted Kaplan-Meier curves and performed univariate Cox analysis, respectively, to further confirm whether or not the OPG has prognostic value.

**Table 1:** Clinicopathological characteristics of patients with hepatocellular carcinoma following curative resection.

Variables	Total (n = 386)
**Age, year, median (range)**	53.00 (42.00–81.00)
**Gender, n (%)**	
Female	50 (12.95)
Male	336 (87.05)
**ALT, U/L, median (range)**	31.50 (8.00–615.00)
**TB, μmol/L, median (range)**	12.30 (3.30–29.00)
**Albumin, g/L, median (range)**	40.91 (31.00–49.00)
**AFP, ng/ml, n (%)**	
Negative	269 (69.69)
Positive	117 (30.31)
**GGT, U/L, n (%)**	
≤50	186 (48.19)
>50	200 (51.81)
**Cirrhosis, n (%)**	
No	110 (28.5)
Yes	276 (71.5)
**HBsAg, n (%)**	
Negative	44 (11.40)
Positive	342 (88.60)
**Size, n (%)**	
≤5	234 (60.62)
>5	152 (39.38)
**Number, n (%)**	
Solitary	324 (83.94)
Multiple	62 (16.06)
**Microvascular invasion, n (%)**	
Absence	264 (68.39)
Presence	122 (31.61)
**Tumor capsule, n (%)**	
None	250 (64.77)
Complete	136 (35.23)
**Differentiation, n (%)**	
I-II	249 (64.51)
III-IV	137 (35.49)
**TNM, n (%)**	
I	234 (60.63)
II	97 (25.13)
III	55 (14.24)
**OPG, ng/ml, median (range)**	4,034.97 (3,412.80–4,848.91)
**TACE, n (%)**	
No	234 (60.62)
Yes	152 (39.38)

ALT, alanine aminotransferase; TB, total bilirubin; AFP, α-fetoprotein; HBsAg, hepatitis B surface antigen; GGT, γ-glutamyltransferase; OPG, Osteoprotegerin; TACE, transcatheter arterial chemoembolization.

Subgroup analyses were also carried out in the unmatched and matched population. The association between serum OPG level and OS or DFS was assessed using Cox multivariate modeling at each subgroup level. Finally, in order to assess the ability of OPG in the prediction of patients’ prognosis in terms of sensitivity or specificity, receiver operating characteristic (ROC) curve was plotted and its corresponding AUC was also calculated. All statistic tests in this study were two-sided, and p<0.05 was set as the threshold of statistical significance. All data analyses were finished using R software (version 3.6.0).

## Results

### Patients’ Characteristics

A total of 386 HCC patients who met our inclusion criteria were enrolled in this study. All of them have undergone radical hepatectomy. As is shown in **Table 1**, most of the patients were male (87.05%, 336/386), while female patients only accounted for 12.95% (50/386). Approximately 81.35% HCC occurred in the background of cirrhosis. The number of HCC patients who carried Hepatitis B was 314 (81.35%). Pathologically confirmed microvascular invasion was detected in 264 HCC patients (68.39%) and absent in 122 patients (31.61%). The majority of patients (232, 20.10%) were at stage I, 96 patients (24.87%) were at stage II, and the rest (58, 15.03%) were at stage III. The detailed description of HCC patients’ clinical characteristics is shown in **Table 1**.

### The Effect of Serum OPG Level on OS and DFS Before Matching

**Figure 1:**
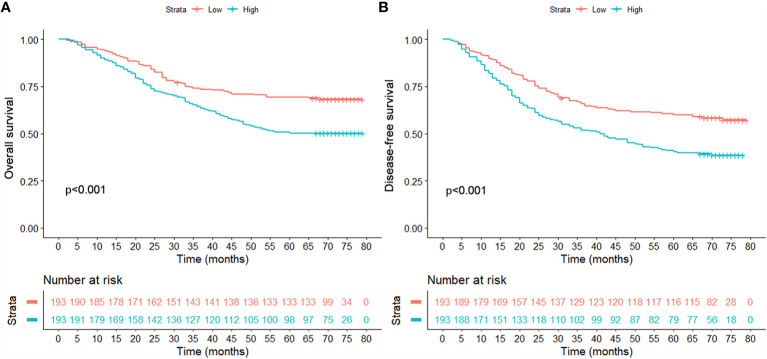
Comparison of overall survival **(A)** and disease-free survival **(B)** between patients with high and low serum levels in the unmatched population.

Though calculation using X-Tile, the optimal cut-off point of OPG was 4,448.58 ng/ml, and the patients were divided into two groups according to this value. There were 240 patients in OPG low group and 146 patients in OPG high group. The univariate cox analysis showed that AFP, GGT, number, MVI, differentiation, TNM stage, OPG, and TACE were strongly correlated with OS and DFS (**Table 2**). Then the above significant variables were incorporated into the Cox proportional hazards multivariate regression model. We found that for OS, AFP, tumor number, microvascular invasion, TNM, and serum OPG level still had prognostic significance. While for DFS, microvascular invasion, TNM, and serum OPG concentration kept statistical significance. Specifically, patients with high OPG levels had poorer OS (HR: 1.93; 95% CI: 1.40–2.66, p<0.001) and DFS (HR: 1.85; 95% CI: 1.39–2.47, p<0.001) than those with low OPG levels. In addition, Kaplan-Meier curves also suggested that serum OPG levels were inversely associated with survival outcomes (**Figure 1**). We also calculated the RMST for each group. The result showed that patients with high OPG had an RMST of 49.60 months (95% CI: 44.98–54.22), while those with low OPG had an RMST of 62.25 months (95% CI: 58.96–65.55). The RMST ratio turned out to be 0.797 (95% CI: 0.716–0.887, p<0.001), suggesting high OPG had negative effects on patients’ prognosis. Similarly, after adjustment for the covariates, the RMST ratio was 0.775 (95% CI: 0.697–0.842, p<0.001).

**Table 2:** Univariate and multivariate Cox regression analysis for assessing the effect of different clinical variables on HCC patients’ overall survival and disease-free survival in the unmatched population.

Variables	Overall survival			Disease-free survival		
	Univariate p	Multivariate analysis		Univariate p	Multivariate analysis	
		HR (95% CI)	p		HR (95% CI)	p
**Age** (>60 *vs.* ≤60*)	0.716			0.333		
**Gender** (male *vs.* female*)	0.356			0.303		
**ALT, U/L** (>50 *vs.* ≤50*)	0.338			0.060		
**TB** (>20 *vs.* ≤20*)	0.411			0.403		
**Albumin** (>40 *vs.* ≤40*)	0.463			0.896		
**AFP, ng/ml** (>400 *vs.* ≤400*)	<0.001	1.57 (1.11–2.21)	0.010	0.004	1.30 (0.96–1.77)	0.091
**GGT, U/L** (>50 *vs.* ≤50*)	<0.001	1.36 (0.97–1.91)	0.073	<0.001	1.29 (0.96–1.73)	0.091
**Cirrhosis** (yes *vs.* no*)	0.184			0.157		
**HBsAg** (positive *vs.* negative*)	0.220			0.298		
**Number** (multiple *vs.* solitary*)	<0.001	1.56 (1.05–2.3)	0.026	0.008	1.26 (0.87–1.81)	0.220
**Microvascular invasion** (presence *vs.* absence*)	<0.001	1.34 (0.92–1.93)	0.123	<0.001	1.23 (0.88–1.72)	0.215
**Tumor capsule** (complete *vs.* none*)	0.419			0.892		
**Differentiation** (III-IV *vs.* I-II*)	0.003	1.36 (0.98–1.89)	0.070	0.006	1.33 (0.99–1.79)	0.056
**TNM**						
II *vs.* I*	<0.001	1.54 (1.03–2.29)	0.035	0.003	1.37 (0.96–1.95)	0.083
III *vs.* I*	<0.001	1.64 (1.04–2.59)	0.034	<0.001	1.64 (1.08–2.49)	0.020
**OPG, ng/ml** (high *vs.* low*)	<0.001	1.93 (1.40–2.66)	<0.001	<0.001	1.85 (1.39–2.47)	<0.001
**TACE** (yes *vs.* no*)	0.032	1.47 (0.92–1.63)	0.061	0.044	1.42 (0.83–1.71)	0.072

ALT, alanine aminotransferase; TB, total bilirubin; AFP, α-fetoprotein; HBsAg, hepatitis B surface antigen; GGT, γ-glutamyltransferase; OPG, Osteoprotegerin; TACE, transcatheter arterial chemoembolization.

**Figure 2:**
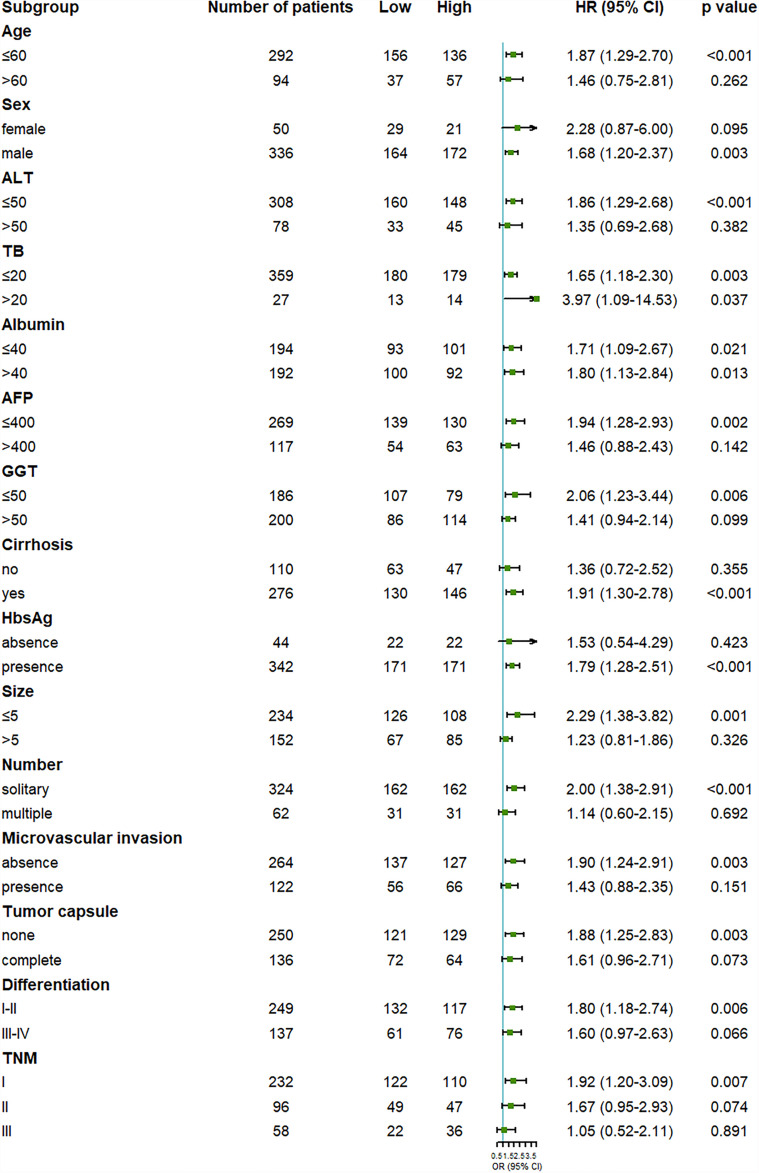
Forest plot showing the effect of serum OPG levels on overall survival in HCC patients stratified by different clinicopathological factors before matching. HR>1 and p<0.05 suggested that higher OPG levels in serum were associated with poorer survival outcome.

**Figure 3:**
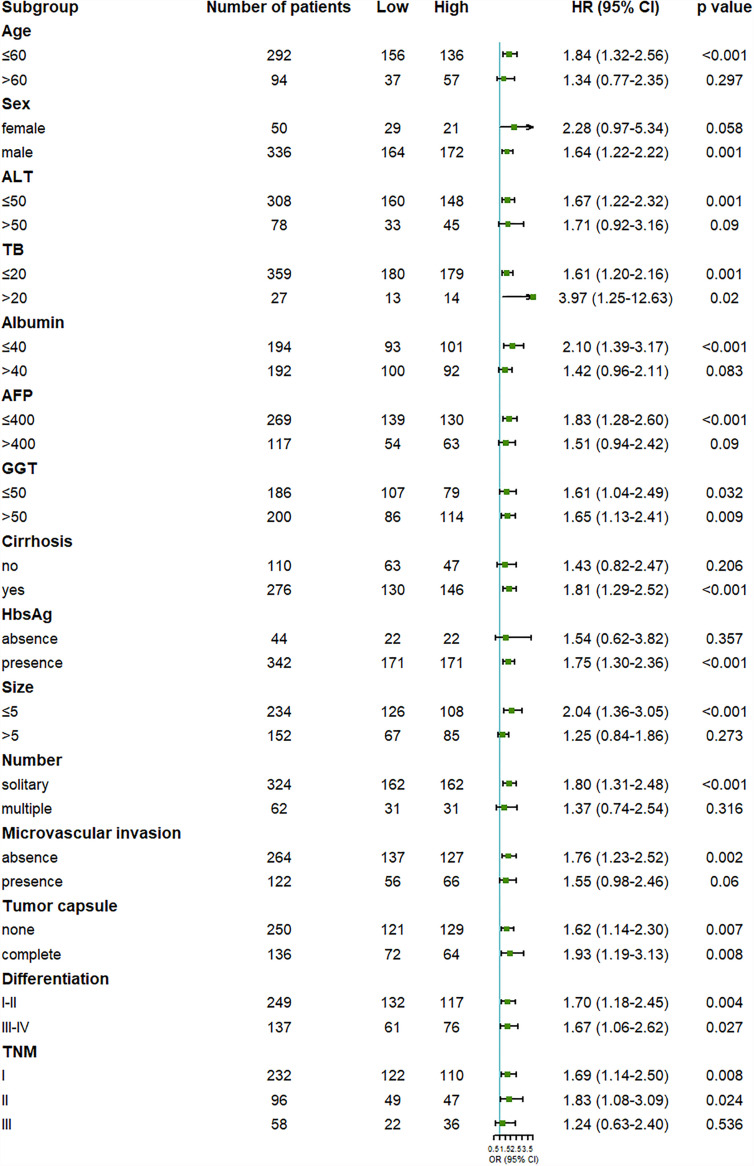
Forest plot showing the effect of serum OPG levels on disease-free survival in HCC patients stratified by different clinicopathological factors before matching. HR>1 and p<0.05 suggested that higher OPG levels in serum were associated with poorer survival outcome.

To further examine the reliability of the results, subgroup analysis was carried out. We found that the outcome of the high OPG group was worse than the low OPG group in most subgroup analyses, even though no statistical significance was observed in some cases (**Figures 2**, **3**). For example, OPG level had no effect on the prognosis of patients who are over 60, which may be due to the limited sample size.

### The Effect of Serum OPG Level on OS and DFS After Matching

To reduce the influence brought by imbalanced covariates, PSM was performed at 1:1 fixed ratio, and finally we got 144 new matched pairs. Standard difference (SD) was used to assess whether or not the matching was eligible. Here, it has shown that the clinical factors between two groups were well balanced with the cut-off of SD as 0.1 (**Table 3**).

**Table 3:** The distribution of clinicopathological variables with standardized difference before and after propensity score matching.

Variables	Before matching		*SD*	After matching		SD
	low (n = 240)	high (n = 146)		low (n = 118)	high (n = 118)	
**Age, n (%)**			0.240			0.057
≤60	191 (79.58)	101 (69.18)		84 (71.19)	87 (73.73)	
>60	49 (20.42)	45 (30.82)		34 (28.81)	31 (26.27)	
**Gender, n (%)**			0.097			<0.001
Female	34 (14.17)	16 (10.96)		14 (11.86)	14 (11.86)	
Male	206 (85.83)	130 (89.04)		104 (88.14)	104 (88.14)	
**ALT, n (%)**			0.23			0.021
≤50	200 (83.33)	108 (73.97)		94 (79.66)	93 (78.81)	
>50	40 (16.67)	38 (26.03)		24 (20.34)	25 (21.19)	
**TB, n (%)**			0.034			0.033
≤20	224 (93.33)	135 (92.47)		110 (93.22)	109 (92.37)	
>20	16 (6.67)	11 (7.53)		8 (6.78)	9 (7.63)	
**Albumin, n (%)**			0.124			0.017
≤40	115 (47.92)	79 (54.11)		61 (51.69)	60 (50.85)	
>40	125 (52.08)	67 (45.89)		57 (48.31)	58 (49.15)	
**AFP, n (%)**			0.113			0.054
Negative	172 (71.67)	97 (66.44)		77 (65.25)	80 (67.80)	
Positive	68 (28.33)	49 (33.56)		41 (34.75)	38 (32.20)	
**GGT, n (%)**			0.367			0.017
≤50	132 (55.00)	54 (36.99)		54 (45.76)	53 (44.92)	
>50	108 (45.00)	92 (63.01)		64 (54.24)	65 (55.08)	
**Cirrhosis, n (%)**			0.214			0.056
No	77 (32.08)	33 (22.60)		35 (29.66)	32 (27.12)	
Yes	163 (67.92)	113 (77.40)		83 (70.34)	86 (72.88)	
**HBsAg, n (%)**			0.022			0.025
Negative	28 (11.67)	16 (10.96)		16 (13.56)	15 (12.71)	
Positive	212 (88.33)	130 (89.04)		102 (86.44)	103 (87.29)	
**Number, n (%)**			0.076			0.070
Solitary	204 (85.00)	120 (82.19)		98 (83.05)	101 (85.59)	
Multiple	36 (15.00)	26 (17.81)		20 (16.95)	17 (14.41)	
**Microvascular invasion, n (%)**			0.067			0.092
Absence	167 (69.58)	97 (66.44)		84 (71.19)	79 (66.95)	
Presence	73 (30.42)	49 (33.56)		34 (28.81)	39 (33.05)	
**Tumor capsule, n (%)**			0.056			0.018
None	153 (63.75)	97 (66.44)		81 (68.64)	80 (67.80)	
Complete	87 (36.25)	49 (33.56)		37 (31.36)	38 (32.20)	
**Differentiation, n (%)**			0.096			0.070
I-II	159 (66.25)	90 (61.64)		77 (65.25)	73 (61.86)	
III-IV	81 (33.75)	56 (38.36)		41 (34.75)	45 (38.14)	
**TNM, n (%)**			0.198			0.036
I	156 (65.00)	78 (53.42)		74 (62.71)	72 (61.02)	
II	63 (26.25)	34 (23.29)		30 (25.42)	31 (26.27)	
III	21 (8.75)	34 (23.29)		14 (11.86)	15 (12.71)	
**TACE, n (%)**			0.065			0.031
No	147 (61.25)	87 (59.59)		69 (58.47)	71 (60.17)	
Yes	93 (38.75)	59 (40.41)		49 (41.53)	47 (39.83)	

ALT, alanine aminotransferase; TB, total bilirubin; AFP, α-fetoprotein; HBsAg, hepatitis B surface antigen; GGT, γ-glutamyltransferase; OPG, Osteoprotegerin; TACE, transcatheter arterial chemoembolization.

**Figure 4:**
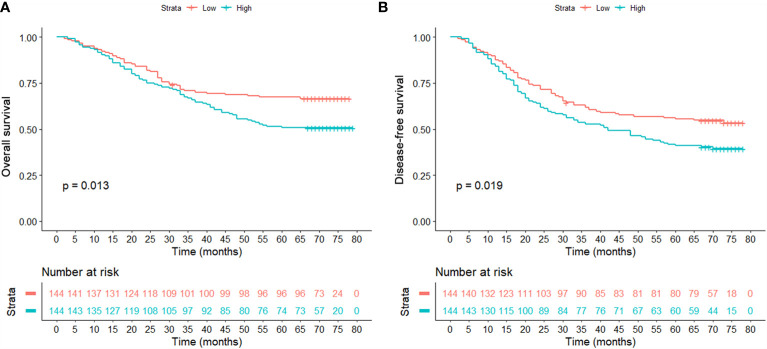
Comparison of overall survival **(A)** and disease-free survival **(B)** between patients with high and low serum levels in the matched population.

After finishing matching, univariate Cox analysis was conducted again to investigate the association between OPG and HCC patients’ prognosis. We found that OPG at higher level still contributed to worse OS (HR: 1.85; 95% CI: 1.25–2.74, p=0.002) and DFS (HR: 1.71; 95% CI: 1.20–2.44, p=0.003) (**Table 4**). Similarly, Kaplan-Meier curves also showed patients’ survival could benefit from low OPG level in terms of OS and DFS (**Figure 4**). As we know, we have to endure the loss of some samples if we conducted PSM analysis, which is thought to be a limitation of PSM method. Due to this reason, half of the subgroup analysis results after PSM indicated OPG was a negative prognostic factor (**Figures S1** and **S2**). But fortunately, none of the analyses suggested OPG was beneficial for OS or DFS.

**Table 4:** Univariate Cox regression analysis for assessing the effect of different clinical variables on HCC patients’ overall survival and disease-free survival in the matched population.

Variables	Overall survival		Disease-free survival	
	Univariate analysis		Univariate analysis	
	HR (95% CI)	p	HR (95% CI)	p
**Age** (>60 *vs.* ≤60*)	0.96 (0.63–1.47)	0.864	1.04 (0.71–1.51)	0.849
**Gender** (male *vs.* female*)	1.19 (0.64–2.22)	0.587	1.11 (0.64–1.94)	0.704
**ALT, U/L** (>50 *vs.* ≤50*)	1.19 (0.76–1.87)	0.443	1.31 (0.87–1.96)	0.202
**TB** (>20 *vs.* ≤20*)	1.44 (0.75–2.76)	0.275	1.52 (0.82–2.83)	0.182
**Albumin** (>40 *vs.* ≤40*)	0.87 (0.59–1.27)	0.469	1.02 (0.72–1.45)	0.895
**AFP, ng/ml** (>400 *vs.* ≤400*)	1.47 (0.99–2.18)	0.003	1.27 (0.89–1.82)	0.186
**GGT, U/L** (>50 *vs.* ≤50*)	1.33 (0.91–1.96)	0.143	1.37 (0.96–1.94)	0.082
**Cirrhosis** (yes *vs.* no*)	1.47 (0.93–2.32)	0.095	1.31 (0.88–1.95)	0.187
**HBsAg** (positive *vs.* negative*)	1.83 (0.92–3.62)	0.083	1.48 (0.84–2.63)	0.178
**Number** (multiple *vs.* solitary*)	1.61 (1.01–2.58)	0.046	1.39 (0.89–2.17)	0.143
**Microvascular invasion** (presence *vs.* absence*)	1.93 (1.31–2.85)	<0.001	1.72 (1.20–2.46)	0.003
**Tumor capsule** (complete *vs.* none*)	1.16 (0.78–1.74)	0.467	1.08 (0.74–1.56)	0.700
**Differentiation** (III-IV *vs.* I-II*)	1.46 (0.99–2.15)	0.054	1.31 (0.92–1.87)	0.134
**TNM**				
II *vs.* I*	1.86 (1.21–2.87)	0.005	1.73 (1.16–2.57)	0.007
III *vs.* I*	2.58 (1.53–4.35)	<0.001	2.38 (1.46–3.88)	<0.001
**OPG, ng/ml** (high *vs.* low*)	1.85 (1.25–2.74)	0.002	1.71 (1.20–2.44)	0.003
**TACE** (yes *vs.* no*)	1.28 (0.73–1.81)	0.191	1.22 (0.69–1.76)	0.203

ALT, alanine aminotransferase; TB, total bilirubin; AFP, α-fetoprotein; HBsAg, hepatitis B surface antigen; GGT, γ-glutamyltransferase; OPG, Osteoprotegerin; TACE, transcatheter arterial chemoembolization.

### Assessment of the Performance of the Model With or Without OPG

**Figure 5:**
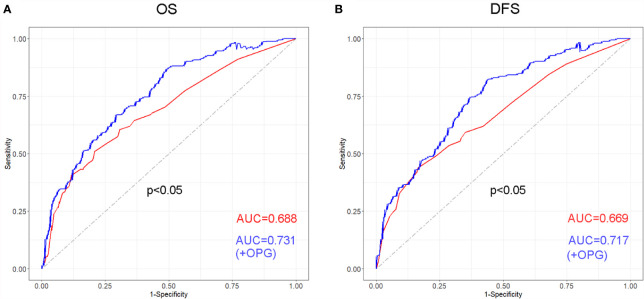
Receiver operating characteristic (ROC) curve and its corresponding AUCs for the models with or without OPG in terms of OS **(A)** and DFS **(B)**.

In order to evaluate discriminative ability of the model with or without OPG, ROC curves were drawn for OS and DFS, respectively (**Figure 5**). We found that for OS, the AUC was 0.731 and 0.688 in the model with and without OPG (p<0.05). While for DFS, the AUC was 0.717 and 0.669 in the model with and without OPG (p<0.05). According to the above result, it is obvious that OPG could increase the sensitivity and specificity of the model for survival prediction.

### Prognostic Comparison Between OPG and AFP, GGT

**Figure 6:**
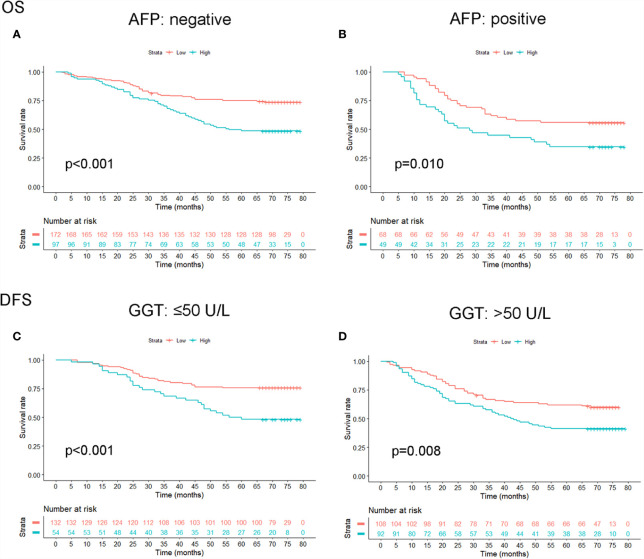
The effect of OPG on HCC patients’ OS **(A, B)** and DFS **(C, D)** stratified by AFP and GGT.

**Figure 7:**
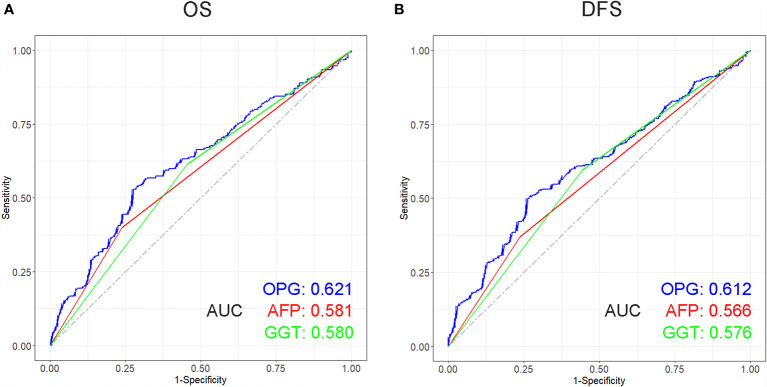
Receiver operating characteristic (ROC) curve of OPG, AFP and GGT in terms of OS **(A)** and DFS **(B)** and its corresponding AUCs.

The prognostic role of OPG was evaluated among patients stratified by AFP and GGT. The result showed that even patients having similar AFP or GGT showed different survival time, which means AFP and GGT had some limitations in the prediction of HCC patients’ prognosis (**Figure 6**). Whereas high OPG still kept having a worse impact on patients’ survival. Through ROC analysis, we found that OPG had larger AUC than AFP and GGT in terms of OS and DFS (**Figure 7**), which suggested that OPG had better discriminative ability of prognostic prediction.

## Discussion

OPG, as a decoy receptor of RANKL, was firstly known as a regulator of bine turnover. Later it was found to be secreted by multiple tumor cells. It has been reported that OPG participated in many tumor biological processes. It can downregulate RANK-signaling by neutralizing either soluble or membrane-bound RANKL, which could affect tumor cell activities. As far as we know, OPG has been proved to promote tumor development and progression in several cancers, including colorectal cancer, gastric cancer, non-metastatic breast cancer, and so on. But no evidence exists about the role of serum OPG in patients with HCC. Our study is the first to show higher serum OPG indicating poorer survival outcome in comparison with those with low OPG level. Results after PSM further confirmed that high OPG was associated with worse OS and DFS. This finding is in accordance with previous report provided by Gao and his colleagues, who observed OPG expression was upregulated in HCC cells under the stimulation of hypoxia and may play a role in tumor aggressiveness. A number of studies demonstrated that OPG has the potential to bind to TNF-related apoptosis-inducing ligand (TRAIL), which exerts pro-apoptotic and anticancer roles in multiple cancers (17, 18). By this way, OPG can prevent tumor cell apoptosis and prolong cells’ survival time, which may partly explain the association between OPG and patients’ prognosis. In addition, it was reported that serum OPG levels were elevated in patients with advanced colorectal cancer (CRC) (19). Overexpression of OPG in CRC tissues was closely related with increased risk of tumor relapse and death, which was suggested as a potential prognostic biomarker for CRC patients. Similarly, Luan et al. demonstrated that OPG expression was increased in gastric cancer (GC) tissue and contributed to patients’ unfavorable outcome. Meanwhile, *in vitro* experiments further proved that OPG could activate Wnt/β-catenin signaling in GC cells and further promote GC cell proliferation, migration, and invasion (20). With all of these taken together, OPG functions as an indicator of poor survival prognosis in different tumors.

However, the interesting thing is that OPG displays contradictory biological behavior in other cases. It was reported that RANKL could stimulate HCC cells to own migratory and invasive ability *via* NF-κB signaling (21). Although OPG, as a receptor of RANKL, was not designed in the whole study, theoretically OPG could inhibit RANKL-dependent downstream molecular signaling and decrease the risk of having HCC. Wan et al. found that the migratory ability of GC cells was improved after RANKL treatment verified by *Transwell* experiments. While OPG, the inhibitor of RANKL, was added and significantly attenuated the GC cell migration (22). For rectal cancer, OPG concentration in serum was elevated during neoadjuvant therapy, and this kind of alteration was associated with a more favorable prognosis (23). It was hypothesized that immune effector priming was mediated by circulating OPG, leading to improved systemic antitumor immunity.

According to the evidence shown above, it seems that OPG plays a dual role in tumors: protumor and antitumor effect. But we should be careful about this conclusion. Because one of the causes is that some of the findings are based on *in vitro* experiments, which just creates a simple and ideal condition and is not the same as the *in vivo* studies. We know that the expression of OPG in the body can be regulated by different kinds of chemical molecules like TRAIL, IL-1β, TGF-β, and PTH. The final inhibitory effect of OPG on RANKL depends on its interaction with these ligands (24, 25).

Given diverse biological functions of OPG in tumor growth, denosumab, as an anti-RANKL monoclonal antibody, was designed to mimic the effect of OPG and prevent skeletal-related events in patients with bone metastases (26). For patients with breast cancer, the fracture risk was decreased after the denosumab administration, and a favorable disease-free survival was also observed in the ABCSG-18 trial (27). In addition, it has been documented that denosumab treatment delayed time to bone metastases in prostate cancer with a median interval of 4.2 months (28). In a similar way, targeted drugs based on the role OPG in HCC might become a new therapeutic approach.

Although several biomarkers, such as AFP and GGT, were reported to have prognostic value in some articles, we found that survival time still differed among patients with similar AFP or GGT levels. Additionally, compared with AFP and GGT, serum OPG showed larger AUC in terms of OS and DFS. The above findings suggested that AFP and GGT had poorer prognostic predictive performance, and OPG may act as a potential novel serum biomarker among HCC patients.

Different from most studies, we adopted ELISA-based method to assess OPG concentration in serum in our study. It has been demonstrated that OPG can be secreted not only by cancer cells but also by cells of the tumor microenvironment. Utilization of ELISA to measure OPG expression in serum could explain the production of OPG from other sources than the tumor cells. Meanwhile, measurement of serum OPG can reduce investigator-related variability. Moreover, immunohistochemical measurement of OPG is based on biopsies from single tumor lesion, which may not be representative considering the fact that OPG expression is not necessarily confined to tumor cells as well as tumor heterogeneity in advanced stage. On the other hand, measurement of serum OPG is relatively easy to perform, less invasive for patients, and can be conducted repeatably compared with immunohistochemical examination.

There are several limitations in our study. First, as this is a retrospective study in essence, potential bias is hard to be avoided even though careful PSM has been conducted. Second, the clinical data is not complete, and some underlying important variables are not available, such as the presence of portal hypertension, but may affect us to accurately estimate the effect of OPG on patients’ prognosis. If these variables could be collected, potential bias would be attenuated to some extent by using PSM analysis. Third, it is better to know serum OPG level in healthy human, which acts as a reference to help us to judge whether OPG concentration is within normal limits. Unfortunately, we only got the OPG levels from HCC patients. Additionally, we have demonstrated that the role of OPG in the prediction of HCC prognosis, but its biological mechanism is not explored in this study. Thus, more basic experiments are urgently needed in order to confirm the functions of OPG in the development of HCC.

## Conclusion

In summary, our study is the first to systematically investigate the effect of serum OPG on HCC patients’ survival outcome. We found that the high level of OPG in serum is associated with worse long-term prognosis. But one thing should be clear that we introduced a novel HCC prognostic biomarker here not aiming to deny the importance value of AFP or other makers in clinical practice, but to provide a new alternative choice for physicians to comprehensively assess patients’ survival and help to determine who can benefit most from treatments.

## Data Availability Statement

The original contributions presented in the study are included in the article/**Supplementary Material**, further inquiries can be directed to the corresponding authors.

## Ethics Statement

This study was approved by the Ethics Committee of Shanghai Ninth People’s Hospital and Zhongshan Hospital. Written informed consent for participation was not required for this study in accordance with the national legislation and the institutional requirements.

## Author Contributions

CZ and JL analyzed the data and wrote the article. XN collected patients’ information. HL, LZ, and ZZ participated in language polish and data check. XQ, HH, and XL were responsible for data visualization. QF and ML designed the study. All authors contributed to the article and approved the submitted version.

## Funding

This study was supported by National Natural Science Foundation of China (Nos. 81770599, 81970526, 81900550) and the Clinical Research Program of Ninth People’s Hospital, Shanghai Jiao Tong University School of Medicine (JYLJ021).

## Conflict of Interest

The authors declare that the research was conducted in the absence of any commercial or financial relationships that could be construed as a potential conflict of interest.

## Publisher’s Note

All claims expressed in this article are solely those of the authors and do not necessarily represent those of their affiliated organizations, or those of the publisher, the editors and the reviewers. Any product that may be evaluated in this article, or claim that may be made by its manufacturer, is not guaranteed or endorsed by the publisher.

## Supplementary Material

Supplementary Figure 1 Forest plot showing the effect of serum OPG levels on overall survival in HCC patients stratified by different clinicopathological factors after matching. HR>1 and p<0.05 suggested that higher OPG levels in serum were associated with poorer survival outcome.

Supplementary Figure 2 Forest plot showing the effect of serum OPG levels on disease-free survival in HCC patients stratified by different clinicopathological factors after matching. HR>1 and p<0.05 suggested that higher OPG levels in serum were associated with poorer survival outcome.
