# Supplementary figures and images for: Prognostic Value of Serum Osteoprotegerin Level in Patients With Hepatocellular Carcinoma Following Surgical Resection
Source: Front Oncol. 2021 Sep 28;11:731989. doi: 10.3389/fonc.2021.731989 (PMC8505987; doi:10.3389/fonc.2021.731989)

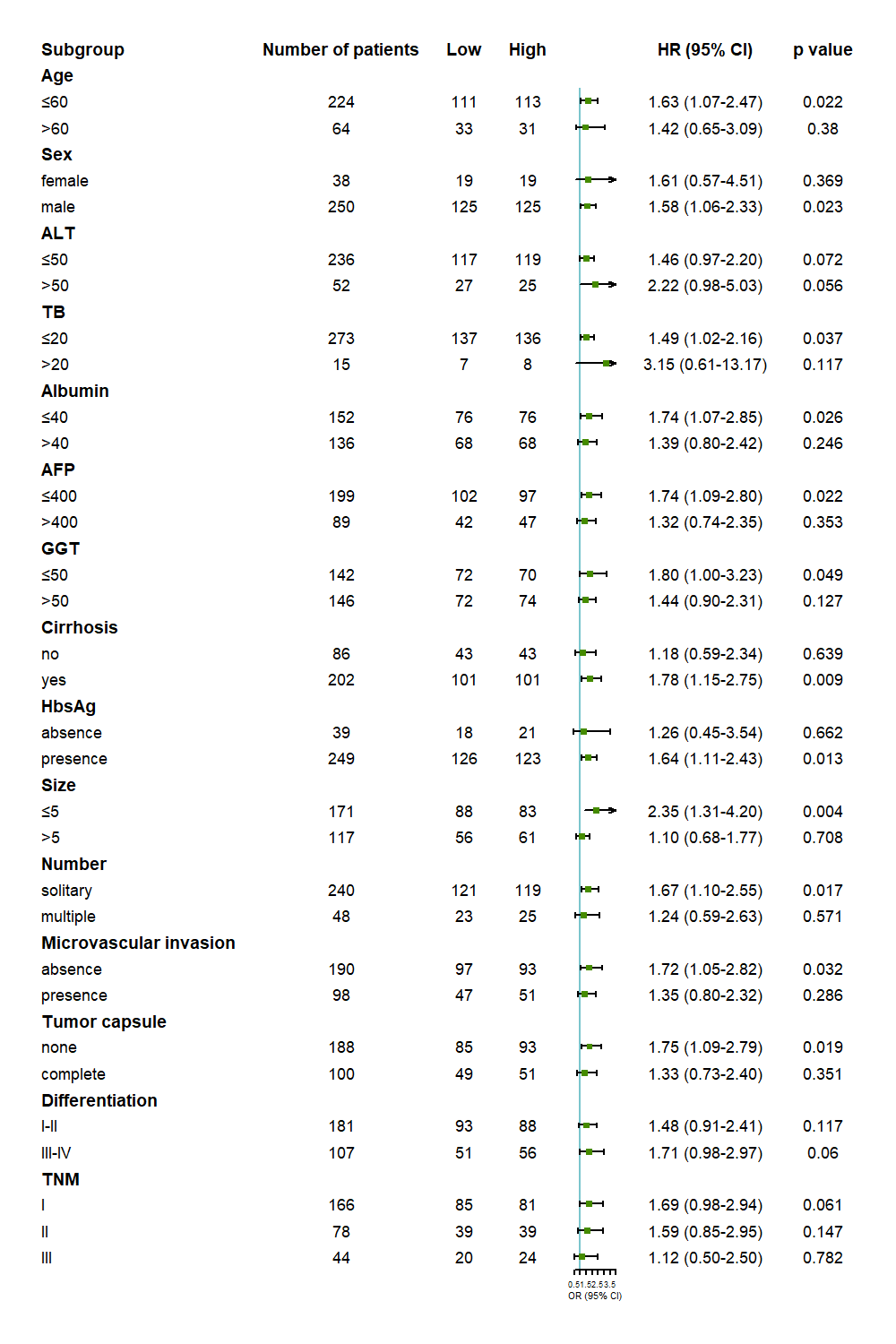

Supplement: Supplementary Figure 1 — Forest plot showing the effect of serum OPG levels on overall survival in HCC patients stratified by different clinicopathological factors after matching. HR>1 and p<0.05 suggested that higher OPG levels in serum were associated with poorer survival outcome. [file Image_1.tiff]

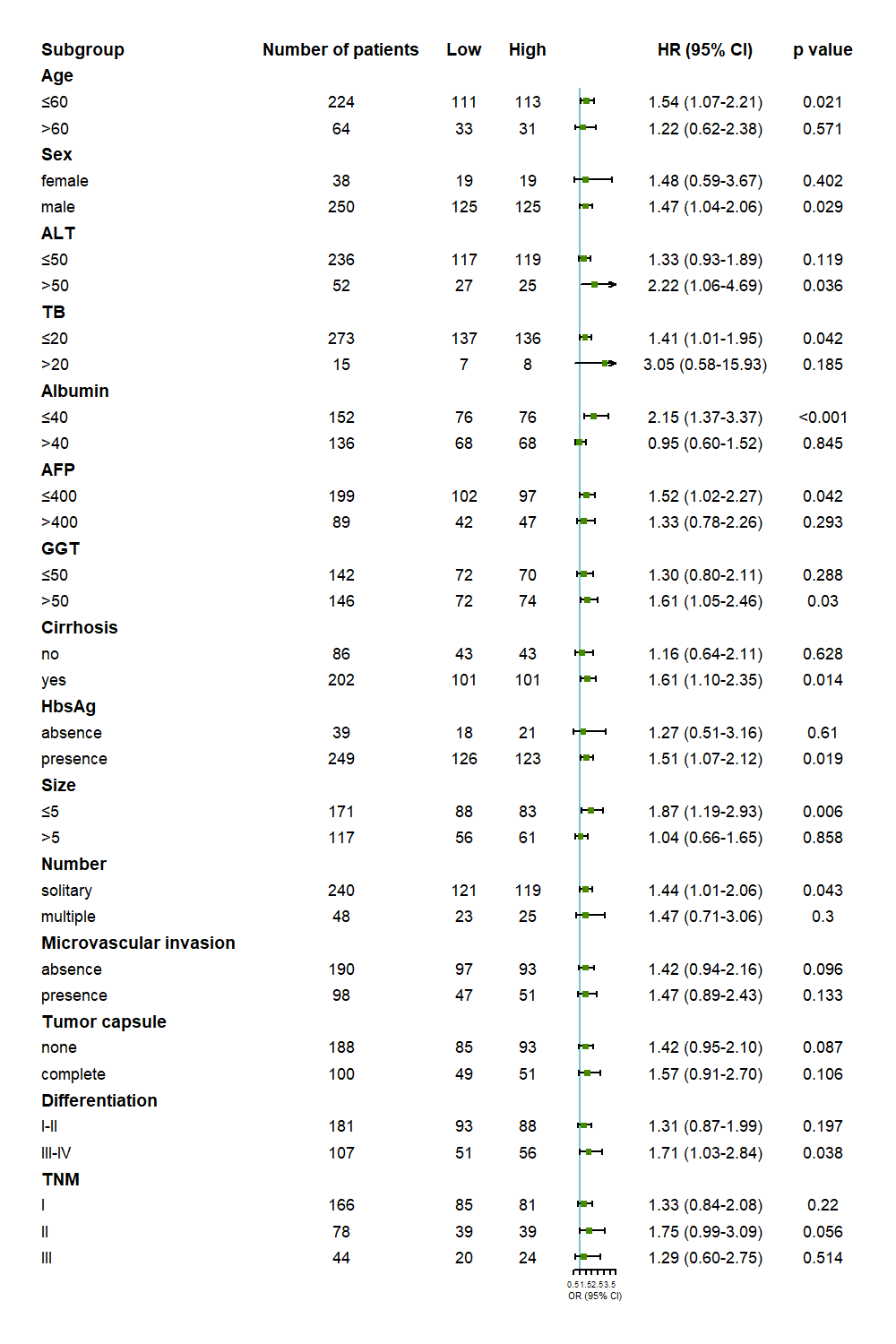

Supplement: Supplementary Figure 2 — Forest plot showing the effect of serum OPG levels on disease-free survival in HCC patients stratified by different clinicopathological factors after matching. HR>1 and p<0.05 suggested that higher OPG levels in serum were associated with poorer survival outcome. [file Image_2.tiff]
